# Variants associated with type 2 diabetes identified by the transethnic meta-analysis study: assessment in American Indians and evidence for a new signal in *LPP*

**DOI:** 10.1007/s00125-014-3351-4

**Published:** 2014-08-12

**Authors:** Anup K. Nair, Yunhua Li Muller, Nellie A. McLean, Maryam Abdussamad, Paolo Piaggi, Sayuko Kobes, E. Jennifer Weil, Jeffrey M. Curtis, Robert G. Nelson, William C. Knowler, Robert L. Hanson, Leslie J. Baier

**Affiliations:** Phoenix Epidemiology and Clinical Research Branch, National Institute of Diabetes and Digestive and Kidney Diseases, National Institutes of Health, 445 North 5th Street, Phoenix, AZ 85004 USA

**Keywords:** American Indians, *ARL15*, *FAF1*, GWAS, *LPP*, *MPHOSPH9*, *POU5F1–TCF19*, *SSR1–RREB1*, Trans-ancestry meta-analysis, Type 2 diabetes

## Abstract

**Aim/hypothesis:**

A recent genome-wide trans-ancestry meta-analysis identified seven new loci associated with type 2 diabetes. We assessed the replication of the seven lead single nucleotide polymorphisms (SNPs) and evaluated these loci for additional signals in American Indians.

**Methods:**

Seven SNPs were genotyped in 7,710 individuals from a longitudinally studied American Indian population, and associations with type 2 diabetes, BMI and related phenotypes were assessed. Previous genome-wide association study (GWAS) data from these individuals were used to screen for additional type 2 diabetes signals at these loci. A variant independent of the trans-ancestry meta-analysis was identified within *LPP*, and its replication was assessed in an additional 3,106 urban American Indians.

**Results:**

SNP rs6813195 near to *TMEM154* was nominally associated with type 2 diabetes (*p* = 0.01, OR 1.12 [95% CI 1.03, 1.22]) and adiposity: the type 2 diabetes risk allele was associated with a lower percentage body fat (β = −1.451%, *p* = 4.8 × 10^−4^). Another SNP, rs3130501 near to *POU5F1–TCF19*, was associated with BMI (β = −0.012, *p* = 0.004), type 2 diabetes adjusted for BMI (*p* = 0.02, OR 1.11 [95% CI 1.02, 1.22]), 2 h glucose concentrations (β = 0.080 mmol/l, *p* = 0.02) and insulin resistance estimated by homeostatic model (β = 0.039, *p* = 0.009). The independent variant identified at the *LPP* locus in our American Indian GWAS for type 2 diabetes was replicated in the additional samples (all American Indian meta-analysis, *p* = 8.9 × 10^−6^, OR 1.29 [95% CI 1.15, 1.45]).

**Conclusions/interpretation:**

For two of the seven newly identified variants, there was nominal evidence for association with type 2 diabetes and related traits in American Indians. Identification of an independent variant at the *LPP* locus suggests the existence of more than one type 2 diabetes signal at this locus.

**Electronic supplementary material:**

The online version of this article (doi:10.1007/s00125-014-3351-4) contains peer-reviewed but unedited supplementary material, which is available to authorised users.

## Introduction

A recent genome-wide transethnic meta-analysis which included European, South and East Asian, Mexican, and Mexican-American populations identified seven new type 2 diabetes susceptibility loci [1]. However, an American Indian population was not included in this meta-analysis. Replication of type 2 diabetes loci identified in earlier genome-wide association studies (GWAS) was assessed in Pima Indians, an American Indian population with a high prevalence of type 2 diabetes, [2–4]. These loci typically had a weaker effect in Pima Indians [2], with the exception of *KCNQ1* (rs2299620), which has a very strong effect in Pima Indians when the risk allele is inherited maternally (*p* = 4.1 × 10^−12^, OR 1.92 per copy of risk allele [95% CI 1.60, 2.31]) [4]. Loci identified in a transethnic cohort may translate across all ethnic groups to a better extent than loci identified in a single ethnic group. Therefore, in the current study we assessed replication in American Indians of the seven lead single nucleotide polymorphisms (SNPs) identified by the transethnic meta-analysis study on type 2 diabetes risk. We also used previous GWAS data from Pima Indians to identify additional SNPs at these loci associated with type 2 diabetes.

## Methods

### Study participants and phenotypes

#### American Indians from the Gila River Indian Community

Biennial health examinations, during which BMI was measured and diabetes status was determined using a 75 g OGTT, were performed in a longitudinal cohort comprising individuals ≥5 years of age. A total of 7,710 community members (electronic supplementary material [ESM] Table 1) were assessed, among whom 3,625 were full-heritage Pima and the remaining 4,085 had some degree of American Indian heritage (on average a person’s heritage was two-thirds American Indian). A subset of non-diabetic individuals (*n* = 561; ESM Table 2) were also characterised in our Clinical Research Center for percentage body fat (PFAT), insulin sensitivity and the acute insulin response, as described elsewhere [5, 6].

#### Urban American Indians

An outpatient sample of urban-dwelling American Indians living in or near to Phoenix, Arizona (*N* = 3,106, mean age 36.6 ± 12.9 years, mean BMI 31.5 ± 7.3 kg/m^2^), was recently recruited to a cross-sectional study. All individuals were ≥50% American Indian, and 76% were full-heritage Indian. Prevalence of type 2 diabetes (28%) was determined using ADA 2010 criteria, based on measures of fasting glucose or HbA_1c_ or previous clinical diagnosis.

### SNP selection and genotyping

SNPs identified by the transethnic meta-analysis study were genotyped by TaqMan genotyping assays (Applied Biosystems, Carlsbad, CA, USA). Previous GWAS data from Pima Indians (specifically, all SNPs mapping approximately 1 Mb upstream and downstream of each lead SNP) were evaluated to identify additional variations at any of the transethnic meta-analysis loci that associated with type 2 diabetes in American Indians [7]. A pairwise tagging approach using Haploview (version 4.2, Broad Institute, Cambridge, Massachusetts, USA) identified 46 tag SNPs (minor allele frequency >0.05 and *r* ^*2*^ ≥ 0.8) from whole genome sequencing data of 234 Pima Indians, representing variations 100 kb upstream and downstream of *LPP* (Chr3:187,740 kb–188,808 kb). Tag SNPs were genotyped using the BeadXpress System (Illumina, San Diego, CA, USA). Genotype quality control required call rates of >95%, no deviation from Hardy–Weinberg equilibrium (*p* > 1.0 × 10^−3^) and a discrepancy rate of <2.5% for blind duplicates (>100 for each sample set).

### Statistical analysis

Statistical analyses were performed using SAS (version 9.2, SAS Institute, Cary, NC, USA). Linear regression models were used to assess the association between continuous traits and genotypes (assuming an additive model), with adjustment for covariates as previously described [7]. Type 2 diabetes association results were analysed both before (*N* = 7,710) and after adjustment for BMI (*n* = 6,587) using log_*e*_ of the maximum recorded lifetime BMI as a covariate (Table 1). The seven lead SNPs were previously reported to be associated with type 2 diabetes at genome-wide significance [1]; therefore, in our study we considered a *p* value of ≤0.05 to indicate significant replication. To assess the significance of additional signals at each locus, a locus-specific Bonferroni-corrected *p* value (0.05/number of SNPs analysed at the respective loci) was used. The power of the current study to detect an effect size similar to the transethnic meta-analysis was calculated using the risk allele frequency (RAF) of the SNPs and diabetes prevalence (34.3%) in American Indians. Results for the rs7649407 SNP in *LPP* were combined in the Pima Indian and urban American Indian samples using the inverse variance method.

**Table 1:** Association of seven transethnic SNPs with type 2 diabetes in longitudinally studied participants from the Gila River Indian Community

SNP	Locus^a^	Allele^a^(R/NR)	Longitudinally studied American Indians							Transethnic meta-analysis GWAS^a^			Power^f^ (*N* = 7,710)
			RAF	T2D^b^ (*N* = 7,710)		BMI^c^ (*N* = 6,839)		T2D (adjmaxBMI)^d^ (*n* = 6,587)		T2D			
				OR (95% CI)^a^	*p*	β	*p*	OR (95% CI)	*p*	RAF^a,e^	OR (95% CI)	*p*	
rs17106184	*FAF1*	G/A	0.992	1.29 (0.78, 2.14)	0.32	0.003	0.88	1.24 (0.75, 2.06)	0.4	0.58–0.95	1.10 (1.07, 1.14)	4.1 × 10^−9^	0.11
rs6808574	*LPP*	C/T	0.966	0.90 (0.67, 1.21)	0.49	−0.025	0.06	0.93 (0.68, 1.27)	0.63	0.60–0.98	1.07 (1.04, 1.09)	5.8 × 10^−9^	0.18
rs6813195	*TMEM154*	C/T	0.53	1.12 (1.03, 1.22)	0.01	−0.007	0.08	1.13 (1.03, 1.23)	0.007	0.39–0.71	1.08 (1.06, 1.10)	4.1 × 10^−14^	0.91
rs702634	*ARL15*	A/G	0.963	0.87 (0.67, 1.14)	0.31	−0.003	0.82	0.88 (0.66, 1.15)	0.35	0.67–0.86	1.06 (1.04, 1.09)	6.9 × 10^−9^	0.16
rs9505118	*SSR1–RREB1*	A/G	0.56	1.08 (0.99, 1.18)	0.08	0.001	0.78	1.08 (0.99, 1.18)	0.1	0.35–0.75	1.06 (1.04, 1.08)	1.4 × 10^−9^	0.71
rs3130501	*POU5F1–TCF19*	G/A	0.63	1.08 (0.99, 1.18)	0.08	−0.012	0.004	1.11 (1.02, 1.22)	0.02	0.43–0.76	1.07 (1.04, 1.09)	4.2 × 10^−9^	0.8
rs4275659	*MPHOSPH9*	C/T	0.47	0.99 (0.90, 1.08)	0.77	0.005	0.28	0.99 (0.91, 1.08)	0.82	0.53–0.78	1.06 (1.04, 1.08)	9.5 × 10^−9^	0.73

^a^Data derived from the genome-wide transethnic meta-analysis report [1]

^b^T2D association was analysed in 3,625 full-heritage Pima Indians and 4,085 mixed-heritage American Indians who had some degree of American Indian heritage (on average a person’s heritage was two-thirds American Indian), the *p* value and OR (95% CI) were adjusted for age, sex, birth year, family membership and admixture estimates

^c^Maximum recorded lifetime BMI (age ≥15 years) was used for the BMI association analysis, *p* values were adjusted for age, sex, birth year, family membership and admixture estimates, β coefficients represent the effect on a logarithmic scale per copy of the risk allele

^d^ *p* values and ORs (95% CI) for T2D association were also adjusted for maximum lifetime BMI (adjmaxBMI) in participants who were defined for both T2D and BMI

^e^RAF ranges for the lead SNPs at the respective loci in Europeans, East Asians, South Asians, Mexicans and Mexican Americans

^f^The power of the current study to detect an effect size for T2D similar to the transethnic meta-analysis study calculated using the RAF and diabetes prevalence in American Indians

NR, non-risk allele; R, risk allele based on genome-wide transethnic meta-analysis [1], T2D, type 2 diabetes

### Approval and consent

All studies were approved by the Institutional Review Board of the National Institute of Diabetes and Digestive and Kidney Diseases. Participants gave written informed consent.

## Results and discussion

Association results for seven new transethnic GWAS SNPs for type 2 diabetes in 7,710 American Indians are shown in Table 1. For comparison, data from the transethnic meta-analysis study are also shown. The best evidence for replication was obtained for rs6813195, near *TMEM154* (*p* = 0.01, OR 1.12 [95% CI 1.03, 1.22]; Table 1). The direction and effect size of the association was consistent and comparable with that reported for individual populations within the transethnic meta-analysis study [1]. SNPs were also assessed for association with BMI, and rs3130501 near *POU5F1–TCF19* had a nominal association (*p* = 0.004, β[log_*e*_] = −0.012; Table 1). Analysis of type 2 diabetes association after adjustment for BMI resulted in significant associations of both rs6813195 and rs3130501 with type 2 diabetes (*n* = 6,587, *p* = 0.007, OR 1.13 [95% CI 1.03, 1.23] and *p* = 0.02, OR 1.11 [95% CI 1.02, 1.22], respectively; Table 1). The diabetes risk alleles for both SNPs were associated with a lower BMI in American Indians, as reported for some other diabetes risk alleles in other studies [8, 9]. Three of the SNPs (rs17106184, rs6808574 and rs702634) had a RAF of >0.96 in American Indians; therefore, their non-significant association in the current study may reflect a lack of power (Table 1). Of the two SNPs with powers >70%, association of rs9505118 with type 2 diabetes was directionally consistent with the transethnic meta-analysis study but did not reach significance (*p* = 0.08, OR 1.08 [95% CI 0.99, 1.18]), while rs4275659 was not associated with type 2 diabetes (Table 1).

Among the other traits analysed in non-diabetic individuals, rs6813195 was associated with PFAT (*p* = 4.8 × 10^−4^, β(%) = −1.451; ESM Table 3): the risk allele for type 2 diabetes was associated with lower body fat and lower BMI. The type 2 diabetes risk allele of rs3130501 associated with higher 2 h glucose concentrations during an OGTT (*p* = 0.02, β = 0.080 mmol/l; ESM Table 3) and insulin resistance, as estimated by HOMA-IR (*p* = 0.009; β = 0.039; ESM Table 3). In the subset of individuals who had undergone an inpatient hyperinsulinaemic–euglycaemic clamp, this allele was also associated with lower insulin sensitivity, although not significantly (*p* = 0.07, β[log_10_] = −0.01; ESM Table 3).

**Fig. 1:**
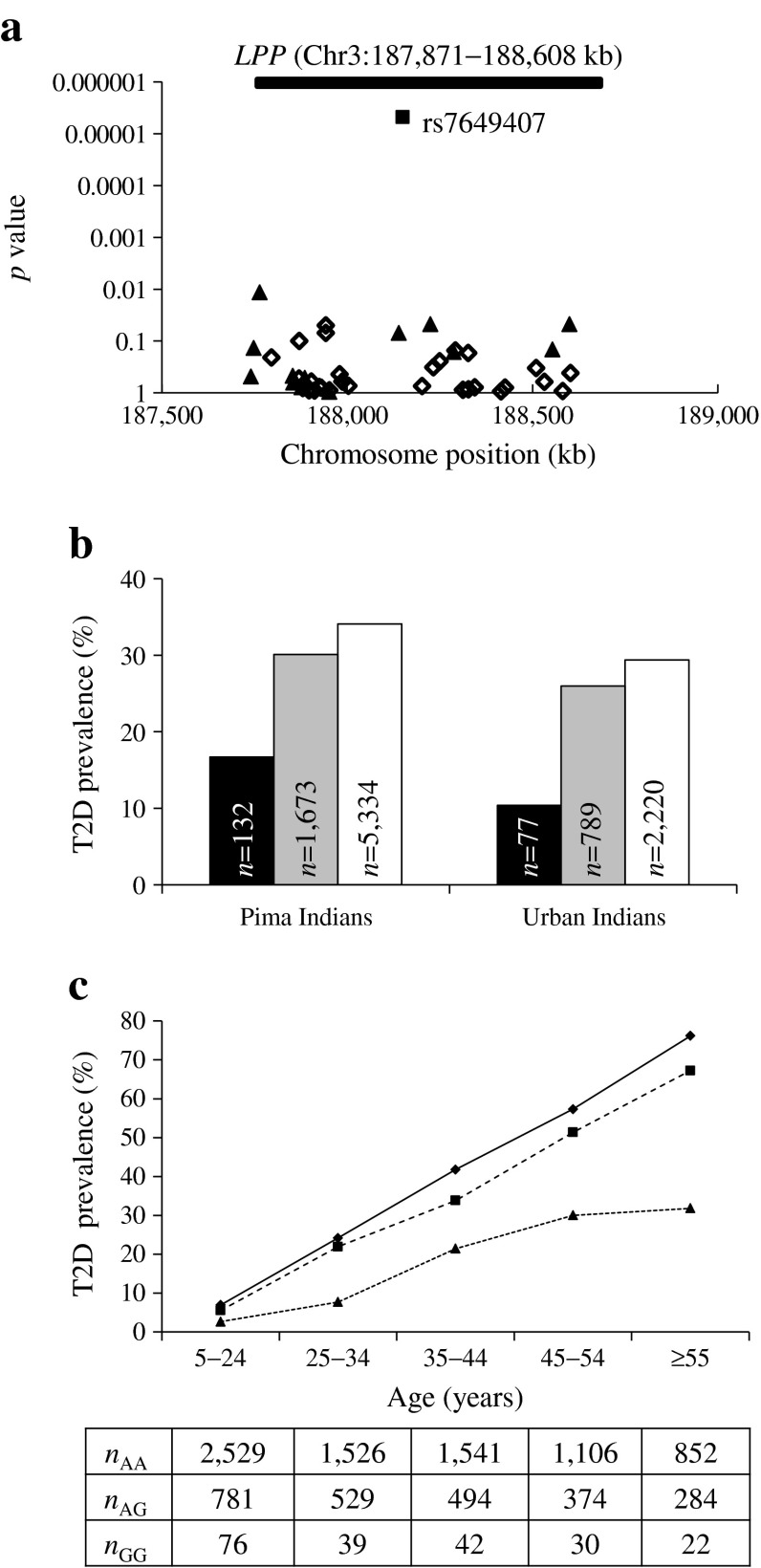
Association of rs7649407 in *LPP* with type 2 diabetes (T2D) in American Indians from the Gila River Indian Community, and replication in cross-sectionally studied urban American Indians. (**a**) Associations of 46 tag SNPs across the region (Chr3:187,740 kb–188,808 kb) encompassing *LPP* with T2D in American Indians. Results were analysed in full-heritage Pima Indians (diamonds; *N* = 3,625), all American Indians from the Gila River Indian community including full-heritage Pima Indians (triangles; *N* = 7,710) and combined American Indians and urban American Indians (squares; *N* = 10,816); *p* values were adjusted for age, sex, birth year, family membership and admixture estimates. (**b**) T2D prevalence in American Indians from the Gila River Indian Community (*p* = 4.1 × 10^−4^, OR 1.29 [1.12, 1.48]) and urban American Indians (*p* = 0.007, OR 1.31 [1.08, 1.59]) by *LPP* SNP rs7649407 (A/G) genotype. All American Indian meta-analysis: *p* = 8.9 × 10^–6^, OR 1.29 [95% CI 1.15, 1.45]. ORs and *p* values were adjusted for age, sex, birth year, family membership and admixture estimates. Black bars, GG genotype; grey bars, AG genotype; white bars, AA genotype. (**c**) T2D prevalence in different age groups by *LPP* SNP rs7649407 genotype. Triangles, GG genotype; squares, AG genotype; diamonds, AA genotype

Evaluation of previous Pima Indian GWAS data at each of the seven loci identified SNPs rs7649407 and rs9882818 (*r* ^*2*^ = 1) at the *LPP* locus (380 SNPs analysed at the *LPP* locus) with evidence of a type 2 diabetes association (*p* = 1 × 10^−4^). As part of our previous GWAS follow up, rs7649407 had been genotyped in additional samples (*N* = 6,834). It was among the top signals for type 2 diabetes and also associated with 2 h glucose levels following an OGTT (*p* = 0.007, β = 0.137 mmol/l) [7]. Therefore, in the current study this locus was investigated further by genotyping 46 tag SNPs that covered common variation across *LPP* in 3,625 full-heritage Pima Indians (ESM Table 4, Fig. 1a). In addition, 13 tag SNPs that nominally associated with type 2 diabetes or BMI were further genotyped in the remaining 4,085 longitudinally studied American Indians (ESM Table 4, Fig. 1a). SNPs tagged by the GWAS SNP (rs7649407) had the strongest association with type 2 diabetes (*p* = 4.1 × 10^−4^, OR 1.29 [95% CI 1.12, 1.48]; Fig. 1b).

To assess the replication of this association, rs7649407 was genotyped in an additional sample of 3,106 urban American Indians. Among these individuals, rs7649407 had a directionally consistent association with type 2 diabetes (*p* = 0.007, OR 1.31 [95% CI 1.08, 1.59]; Fig. 1b), and a meta-analysis combining the two samples showed a stronger association (*p* = 8.9 × 10^−6^, OR 1.29 [95% CI 1.15, 1.45], Fig. 1a, b). The association was consistent across all age groups (Fig. 1c). This SNP (rs7649407) exhibits low linkage disequilibrium with rs6808574 (near to *LPP*), which was reported in the transethnic meta-analysis study (*D′* = 0.14, *r* ^2^ = 0 in Pima Indians; *D′* = 0.08, *r* ^2^ = 0 in Utah residents with ancestry from northern and western Europe (CEU); HapMap V3, R2, http://hapmap.ncbi.nlm.nih.gov/). In American Indians, the association of rs7649407 was unaffected by a conditional analysis with rs6808574. Therefore, we propose that the *LPP* locus may contain more than one region of association with type 2 diabetes. It is noteworthy that rs7649407, with a RAF of 0.88 in the present study, has a RAF of 0.99 in some Asian populations and may therefore not be informative in these populations. ENCODE data shows that rs7649407 maps to an intronic region with a strong enhancer effect in multiple cell types, including skeletal muscle myoblasts [10]. However, we did not find a clear relationship between rs7649407 genotype and *LPP* expression in 250 skeletal muscle biopsies from Pima Indians (data not shown). The role of the encoded protein, lipoma-preferred partner (LPP), in the regulation of glucose metabolism is unknown and will require further investigations.

In conclusion, analysis of type 2 diabetes SNPs initially reported in a transethnic meta-analysis identified nominal replication of two SNPs (rs6813195 and rs3130501) in Americans Indians. Analysis of the implicated loci in this population of non-European ancestry identified an independent *LPP* variant that reproducibly associated with type 2 diabetes in American Indians, thus demonstrating the utility of including populations of diverse ‘nonstandard’ ancestry in the identification of susceptibility loci.

## Electronic supplementary material

Below is the link to the electronic supplementary material.

ESM Table 1 (PDF 163 kb)

ESM Table 2 (PDF 125 kb)

ESM Table 3 (PDF 19.3 kb)

ESM Table 4 (PDF 177 kb)

## Abbreviations

GWAS: Genome-wide association study
PFAT: Percentage body fat
RAF: Risk allele frequency
SNP: Single nucleotide polymorphism
