# Supplementary material for: Variants associated with type 2 diabetes identified by the transethnic meta-analysis study: assessment in American Indians and evidence for a new signal in LPP
Source: Diabetologia. 2014 Aug 12;57(11):2334–8. doi: 10.1007/s00125-014-3351-4 (PMC4180905; doi:10.1007/s00125-014-3351-4)
Supplement: Supplementary file 1 — (PDF 163 kb) [file 125_2014_3351_MOESM1_ESM.pdf]

ESM Table 1: Characteristics of subjects from the Gila River Indian Community.

Longitudinally studied subjects from Gila River Indian Community	
	Diabetes analysis set (*N*=7,710)
Diabetic (%M)/non diabetic(%M)	2549 (38.5%)/5161 (47.4%)
Age in years (diabetic/non diabetic)	46.5 ± 14.6/ 27.6 ± 13.4
BMI (kg/m^2)	38.7 ± 8.6/ 34.6 ± 8.5
	BMI analysis set (*n*=6,839)[a]
Age in years	32.7 ± 13.2
BMI (kg/m^2)	36.1 ± 8.8
	Metabolic phenotypes analyzed (*n*=5,429)[b]
Fasting glucose (mmol/l)	5.3 ± 0.6
Fasting Insulin (SD unit)	-0.025 ± 0.97
2 h-glucose (mmol/l)	6.4 ± 1.8
HOMA-IR(SD unit)	-0.203 ± 0.88
HOMA-B (SD unit)	0.30 ± 0.73

Data are presented as mean ± SD. (%M) - percentage of male subjects. Diabetes analysis set consisted of 3,625 full-heritage Pima Indians and 4,085 mixed-heritage American Indians. [a] Maximum recorded lifetime BMI after age 15 years was used for BMI association analysis. Of 7,710 subjects only 6,839 were defined for BMI and hence were used for the analysis. [b] Metabolic phenotypes used for analysis were from the last visit when the subject was non diabetic. Subjects who were diabetic during the first exam were not included in this analysis and hence only 5,429 out of the 7,710 subjects were included. Because Insulin was measured using different assays over a period of time (longitudinal data), insulin values were converted to z-scores by assay for comparability. HOMA-IR, HOMA-B and fasting insulin were log transformed and are expressed in SD units. HOMA-IR, HOMA-B; homeostatic model assessment of insulin resistance and beta cell function.
