# Supplementary material for: Variants associated with type 2 diabetes identified by the transethnic meta-analysis study: assessment in American Indians and evidence for a new signal in LPP
Source: Diabetologia. 2014 Aug 12;57(11):2334–8. doi: 10.1007/s00125-014-3351-4 (PMC4180905; doi:10.1007/s00125-014-3351-4)
Supplement: Supplementary file 2 — (PDF 125 kb) [file 125_2014_3351_MOESM2_ESM.pdf]

ESM Table 2: Characteristics of metabolically studied non-diabetic subjects from the Gila River Indian Community.

**n (% Male)**	561 (59%)
**Age range (years)**	18–46
**Mean Age (years) ± SD**	26.8 ± 6.1
**Mean BMI (kg/m^2) ± SD**	33.4 ± 7.5
**Mean PFAT (%) ± SD**	32.3 ± 8.5
**Mean log$_{10}$ of glucose disposal rates during insulin infusions (M) (mg·kg EMBS^{a-1} · min^{-1}) ± SD**	0.56 ± 0.12
**Mean log$_{10}$ acute insulin response[b] (μU/ml) ± SD**	2.32 ± 0.28

[a] EMBS= estimated metabolic body size; [b] only full-heritage Pima Indians with normal glucose tolerance (N=301) were analyzed for acute insulin response.
