# Supplementary material for: Variants associated with type 2 diabetes identified by the transethnic meta-analysis study: assessment in American Indians and evidence for a new signal in LPP
Source: Diabetologia. 2014 Aug 12;57(11):2334–8. doi: 10.1007/s00125-014-3351-4 (PMC4180905; doi:10.1007/s00125-014-3351-4)
Supplement: Supplementary file 3 — (PDF 19.3 kb) [file 125_2014_3351_MOESM3_ESM.pdf]

ESM Table 3: Association of SNPs (with mAF>0.05 in American Indians[a]) identified by genome-wide trans-ancestry meta-analysis with measures of adiposity (PFAT), Insulin sensitivity (fasting glucose, fasting Insulin, HOMA-IR and $Log_{10}M$) and Insulin secretion (HOMA-B, $Log_{10}AIR$) in American Indians.

SNP ID<br>Locus<br>Alleles (R/NR)	rs6813195<br>*TMEM154*<br>C/T		rs9505118<br>*SSR1-RREB1*<br>A/G		rs3130501<br>*POU5F1-TCF19*<br>G/A		rs4275659<br>*MPHOSPH9*<br>C/T	
	beta	*P*	beta	*P*	beta	*P*	beta	*P*
PFAT [b,f] (%)	-1.451	4.8×10^{-4}	0.595	0.17	0.335	0.47	-0.37	0.42
Fasting Glucose [c,d] (mmol/l)	-0.013	0.25	-0.007	0.54	0.002	0.87	-0.016	0.16
Log Fasting Insulin [c,d] (SD units)	-0.036	0.07	0.001	0.96	0.016	0.42	0.009	0.63
2 h Glucose [c,d] (mmol/l)	0.049	0.16	0.005	0.87	0.080	0.02	-0.032	0.35
HOMA-IR [c,e](SD units)	-0.013	0.4	-0.007	0.61	0.039	0.009	0.005	0.72
$Log_{10}M$[b,g] (mg·kg EMBS*$^{-1}$ · min^{-1})	0.005	0.35	0.012	0.09	-0.01	0.07	0.007	0.24
HOMA-B[c,e](SD units)	-0.003	0.81	0.001	0.93	0.025	0.06	0.015	0.28
$Log_{10}AIR$[b, h] (μU/ml)	-0.016	0.51	-0.008	0.72	0.02	0.27	0.046	0.06

[a] Number of subjects with metabolic data was relatively small; therefore, analysis was restricted to SNPs with mAF>0.05. [b] PFAT, $Log_{10}M$ and $Log_{10}AIR$ were analyzed in metabolically characterized non-diabetic subjects (*n*=561). [c] Fasting glucose, Fasting Insulin, 2 hr-glucose, HOMA-IR and HOMA-B were analyzed in longitudinally studied subjects (*n*=5,429). [d] *P* values were adjusted for age, sex, birth year, family membership and admixture estimates. [e] *P* values were adjusted for age, sex, birth year, family membership, admixture estimates and BMI. [f] *P* values were adjusted for age, sex and family membership; [g]*P* values were adjusted for age, sex, PFAT and family membership; [h] analysis is restricted to full heritage Pima Indians with normal glucose tolerance and *P* values were adjusted for age, sex, PFAT, family membership and M. Because Insulin was measured using different assays over a period of time (longitudinal data), insulin values were converted to z-scores by assay for comparability. HOMA-IR, HOMA-B and fasting insulin were log transformed and are expressed in SD units. Locus is based on the genome-wide trans-ethnic meta-analysis report [1]. R: risk allele based on genome-wide trans-ethnic meta-analysis [1], NR: non-risk allele, PFAT: percent body fatness, M: glucose disposal rate during insulin infusions, AIR: acute insulin response during glucose infusion.
