# Supplementary material for: Variants associated with type 2 diabetes identified by the transethnic meta-analysis study: assessment in American Indians and evidence for a new signal in LPP
Source: Diabetologia. 2014 Aug 12;57(11):2334–8. doi: 10.1007/s00125-014-3351-4 (PMC4180905; doi:10.1007/s00125-014-3351-4)
Supplement: Supplementary file 4 — (PDF 177 kb) [file 125_2014_3351_MOESM4_ESM.pdf]

ESM Table 4: Association of 46 tag SNPs in *LPP* with T2D in American Indians

		Allele	Full-heritage Pima Indians (N=3,625) T2D			American Indians (full-heritage +mixed-heritage, N=7,710) T2D		
SNP	Location	R/NR	RAF	OR (95% CI)	*P*	RAF	OR (95% CI)	*P*
rs6808574[a]	5'-upstream	C/T	0.99	1.15 (0.46-2.87)	0.76	0.96	0.90 (0.67-1.21)	0.49
rs4686925	5'-upstream	A/G	0.8	1.10 (0.95-1.28)	0.22	0.73	1.08 (0.98-1.20)	0.14
rs2201822	5'-upstream	G/C	0.88	1.17 (0.97-1.42)	0.1	0.87	1.19 (1.04-1.36)	0.01
rs2037184	5'-upstream	C/T	0.71	1.08 (0.96-1.23)	0.21			
rs3913218	5'-upstream	T/C	0.75	1.10 (0.96-1.27)	0.17	0.76	1.04 (0.94-1.15)	0.47
rs12488886	5'-upstream	C/T	0.76	1.07 (0.94-1.22)	0.31	0.73	1.02 (0.93-1.13)	0.63
rs7616349	5'-upstream	C/G	0.32	1.04 (0.92-1.18)	0.53			
rs57280116	5'-upstream	C/T	0.84	1.14 (0.98-1.34)	0.1			
rs10937342	intron	C/A	0.26	1.02 (0.89-1.16)	0.81	0.25	0.99 (0.89-1.09)	0.79
rs4686476	intron	G/A	0.66	1.02 (0.90-1.16)	0.73			
rs12637043	intron	G/A	0.64	1.02 (0.90-1.15)	0.81			
rs9881941	intron	C/A	0.79	1.08 (0.93-1.25)	0.32	0.78	1.04 (0.93-1.15)	0.51
rs13096994	intron	G/A	0.47	1.01 (0.90-1.14)	0.86			
rs4577458	intron	T/G	0.61	1.01 (0.90-1.13)	0.9			
rs932154	intron	C/G	0.94	1.02 (0.80-1.31)	0.86			
rs9857023	intron	C/T	0.94	1.08 (0.82-1.40)	0.61			
rs4686478	intron	C/T	0.54	1.01 (0.89-1.14)	0.91			
rs7645376	intron	A/G	0.25	1.02 (0.90-1.16)	0.78			
rs1842306	intron	C/T	0.71	1.02 (0.89-1.16)	0.8			
rs28403879	intron	C/A	0.68	1.12 (0.99-1.27)	0.07			
rs74698002	intron	G/A	0.14	1.18 (1.00-1.40)	0.05			
rs4381925	intron	T/C	0.54	1.01 (0.90-1.13)	0.89			
rs4449306	intron	A/C	0.68	1.09 (0.97-1.24)	0.15	0.66	1.00 (0.92-1.10)	0.96
rs6784029	intron	G/A	0.79	1.06 (0.92-1.22)	0.44			
rs2306375	intron	G/T	0.82	1.04 (0.90-1.20)	0.61			
rs6777007	intron	A/G	0.43	1.02 (0.91-1.15)	0.73			

		Allele	Full-heritage Pima Indians (N=3,625) T2D			American Indians (full-heritage +mixed heritage, N=7,710) T2D		
SNP	Location	R/NR	RAF	OR (95% CI)	*P*	RAF	OR (95% CI)	*P*
rs6789800	intron	A/G	0.93	1.17 (0.91-1.51)	0.23	0.9	1.16 (0.99-1.37)	0.07
rs7649407	intron	A/G	0.88	1.36 (1.12-1.67)	0.002	0.86	1.29 (1.12-1.48)	$4x10^{-4}$
chr3:188202403	P73S	C/T	0.99	1.09 (0.65-1.82)	0.74			
rs7428265	intron	A/G	0.91	1.26 (1.02-1.55)	0.03			0.07
rs16863396	intron	T/C	0.43	1.06 (0.94-1.20)	0.32	0.9	1.15 (0.99-1.33)	
rs2081550	intron	A/G	0.65	1.08 (0.95-1.22)	0.25			
rs4591495	intron	G/C	0.93	1.24 (0.99-1.44)	0.06	0.9	1.11 (0.96-1.29)	0.16
rs10937356	intron	T/G	0.93	1.17 (0.94-1.47)	0.15			
rs1828952	intron	A/G	0.95	1.02 (0.78-1.33)	0.87			
chr3:188326934	intron	-/T	0.64	1.09 (0.96-1.24)	0.17			
rs1059380	D314D	T/C	0.63	1.01 (0.90-1.14)	0.85			
rs7635459	intron	G/A	0.47	1.02 (0.90-1.15)	0.78			
rs16863539	intron	G/A	0.07	1.01 (0.81-1.27)	0.94			
rs9877192	intron	C/T	0.1	1.03 (0.84-1.25)	0.79			
rs3915010	intron	T/C	0.14	1.09 (0.91-1.30)	0.33			
rs12495456	intron	C/T	0.78	1.04 (0.90-1.20)	0.61			
rs4686494	intron	G/A	0.91	1.12 (0.91-1.38)	0.27	0.86	1.11 (0.97-1.27)	0.15
rs9876347	intron	T/C	0.92	1.01 (0.82-1.25)	0.93			
rs6793623	3'-UTR	T/C	0.56	1.14 (1.01-1.28)	0.03	0.54	1.09 (1.00-1.19)	0.05
rs2378456	3'-UTR	G/C	0.45	1.05 (0.93-1.19)	0.41			

[a] Indicates the type 2 diabetes variant in the trans-ethnic meta-analysis. The risk allele (given first) for rs6808574 is defined as the observed risk allele in this trans-ethnic study, while for other SNPs it is defined as the allele with a higher risk of diabetes in full heritage Pima Indians; ORs are given per copy of this allele. OR and *P* values were adjusted for age, sex, birth year, family membership and admixture estimates. RAF: frequency of the risk allele, T2D: type 2 diabetes.
